# Voice symptoms, dysphagia risk, quality of life, and mental health in total laryngectomized patients: a self-perception study

**DOI:** 10.1016/j.bjorl.2025.101657

**Published:** 2025-06-25

**Authors:** João Vitor Barbosa Pereira, Vaneli Colombo Rossi, Carlos Takahiro Chone, Ana Carolina Constantini

**Affiliations:** a Universidade Estadual de Campinas, Departamento de Desenvolvimento Humano e Reabilitação, Campinas, SP, Brazil; b Universidade Estadual de Campinas, Departamento de Oftalmologia/Otorrinolaringologia, Campinas, SP, Brazil

**Keywords:** Laryngectomy, Voice, Swallowing, Quality of life, Mental health

## Abstract

**Objectives:**

To establish the sociodemographic profile, identify and relate the presence of voice symptoms, risk for dysphagia, and self-perceived emotional distress, and measure the quality of life of a group of total laryngectomized patients.

**Method:**

This is a descriptive, quantitative, cross-sectional study. Subjects aged 18-years and older, of both sexes, who had undergone total laryngectomy. The sample composition was non-probabilistic. A questionnaire was used to obtain sociodemographic data. The perception of voice symptoms was assessed by the Voice Symptom Scale (VoiSS) and the risk of dysphagia was screened using the Eating Assessment Tool (EAT-10). Mental health aspects were measured using the Self-Reporting Questionnaire (SRQ-20) and data on general health, treatment and illness were collected using the European Organisation for Research and Treatment of Cancer Quality of Life Questionnaire Core-30 (EORTC QLQ-C30).

**Results:**

12 participants were recruited; mostly male, average age of 64-years. All participants presented voice symptoms and 33% were screened with self-perceived emotional distress and 66.7% progressed with risk for dysphagia; all of them had undergone at least radiotherapy during the treatment.

**Conclusion:**

All subjects in the sample had voice symptoms. Risk of dysphagia emerged as a determining factor in the quality of life and mental health of study participants, and was also related to concomitant voice symptoms. In this sample, participants with a high prevalence of voice signs and symptoms were at greater risk of emotional distress.

## Highlights

• Total laryngectomy patients self-report vocal symptoms.
• Risk for dysphagia is a determinant of quality of life and mental health.
• The risk for dysphagia is related to greater functional impairment.
• Subjects in psychological distress presented greater functional and health impairment.

## Introduction

Head and neck squamous cell carcinoma affects 2% of the Brazilian population, with laryngeal cancer being the second most common type.^1^, ^2^, ^3^ During treatment, the oncology team assesses the tumor site (glottis, supraglottis, or subglottis), tumor progression, physical conditions, psychosocial aspects, and quality of life.^4^, ^5^

Despite advances in treatment and stable survival rates, all treatment methods affect the quality of life of cancer patients. Surgeries and/or procedures involving chemoradiotherapy have limiting transient or permanent side effects, affecting their daily activities, especially those related to phonation and swallowing, as well as their social and interpersonal relationships.^6^

Patients who have undergone a total laryngectomy lose the ability to communicate through the laryngeal voice.^7^ During swallowing, a prolonged oral and pharyngeal transit time is observed, as well as residues in the oral cavity, a reduced opening time of the cricopharyngeal muscle, and odynophagia, which affects the well-being during eating and, consequently, changes the eating habits of this population, reducing their quality of life, since 80% of laryngectomized subjects cite voice and swallowing aspects as essential in their daily lives.^8^, ^9^, ^10^

Studies show a high prevalence of anxiety and depression in the laryngectomized population.^11^, ^12^ However, the relationship between these health conditions and swallowing has not yet been clearly described in the literature, despite evidence indicating a decline in the quality of life of patients with difficulty swallowing and emotional distress after treatment.^28^ Speech therapy and multidisciplinary interventions are critical to minimize these effects, enable social reintegration, and improve the quality of life of these individuals.^4^

The concept of quality of life refers not only to the absence of illness, but also to the complete physical, mental, and social well-being of individuals,^13^ as well as the degree of satisfaction with family, social, love, and environmental aspects. Therefore, learning more about the factors related to the quality of life and mental health of subjects undergoing cancer treatment leads to the provision of comprehensive health care and a better understanding of the treatment effects on their routine, allowing for improvements in clinical support, rehabilitation, and quality of life.^6^

On the other hand, measuring the quality of life is a challenging task, given the unique understanding of one’s own experiences, socioeconomic variables, and different cultural levels. Despite the undeniable importance of the relationship between voice, swallowing, and quality of life in laryngectomized patients, and the studies conducted in recent years on this topic, there is a lack of data about these aspects and their relationships before and after treatment.^9^

Considering the importance of voice and swallowing for a satisfactory quality of life throughout rehabilitation, as well as the need for a better understanding of this topic in order to improve therapeutic support, this study aimed to outline the sociodemographic profile, identify and correlate the presence of voice symptoms, risk for dysphagia and emotional distress, and quality of life in a group of total laryngectomized individuals.

## Methods

A descriptive, quantitative, cross-sectional study was conducted at the Otorhinolaryngology Outpatient Clinic of the UNICAMP. It was approved by the Institution’s Research Ethics Committee according to report nº 4.799.555. All participants signed an informed consent form. The study included all consecutive individuals aged over 18-years, of both sexes, who had undergone total laryngectomy and were under monitoring at Hospital das Clínicas (HC-UNICAMP) from year to year. This health center is an Otorhinolaryngology/Head and Neck outpatient of a public sector school hospital, considered as a reference in the state of São Paulo, which serves the metropolitan region of Campinas. The service has a multidisciplinary team of otorhinolaryngologists, nurses, speech therapists, and psychologist. Upon admission to the service, participants undergo an assessment by the multidisciplinary team to establish health conduct. Participants from our sample were recruited between April and June 2021, which included the period of the COVID-19 pandemic. During this period, due to the pandemic restrictions only individuals previously under follow-up and who had some medical demand returned for an outpatient appointment, limiting the flow of patients and, consequently, the composition of our sample. The following exclusion criteria were individuals with suspected recurrence or metastasis; neurological diagnoses and/or possible cognitive alterations that prevented participants from understanding or performing the procedures; and individuals who had abandoned the outpatient care.

Eligible participants answered a questionnaire to obtain sociodemographic data and sample characterization, such as sex, age, time since diagnosis, voice rehabilitation method, and treatments performed. The sociodemographic questionnaire and the protocols were applied by the same researcher, who read all the questions and waited for the participant’s answers. During the applications the researcher was available to support the participants regarding any doubts. To obtain voice symptoms, the Voice Symptom Scale (VoiSS) was applied; this protocol was translated into and culturally adapted for Portuguese,^14^ with 30 questions – 15 related to the limitation domain, 8 to the emotional domain, and 7 to the physical domain. All questions have 5 items with the following possible answers: always = 4-points; almost always = 3-points; sometimes = 2-points; rarely = 1-point; and never = 0-point. The scores of each domain are added to obtain a partial score. Then the partial scores of all domains are added to obtain the total sum. The limitation domain has a minimum score of 0 (zero) and a maximum score of 60, while the emotional domain ranges from 0 (zero) to 32 points and the physical domain ranges from zero 0 (zero) to 28. The final score ranges from 0 (zero) to 120, with higher VoiSS scores indicating stronger perception of voice deviation. This instrument presents 100% sensitivity and specificity in cases of organic dysphonia due to head and neck cancer.^15^

The risk of dysphagia was measured using the Eating Assessment Tool (EAT-10), which was translated into and culturally adapted for Portuguese.^16^ It holds 10 questions – 3 related to the functional domain, 3 to the emotional domain, and 4 to the physical and organic domain. The final score ranges from 0 (zero) to 40, with a score higher than 3 indicating risk of dysphagia.

The Self-Reporting Questionnaire (SRQ-20) with 20 items^17^ was developed by the World Health Organization and adapted for the Brazilian population^18^ to assess Common Mental Disorders (CMD) and track psycho-emotional disorders. In our study, this instrument helped track individuals with emotional distress. It holds 20 questions, with yes/no answers, and one point added for each affirmative answer. A score equal to or above 8 indicates suspicion of psychological morbidity. Participants who exceeded this score were referred to the psychology care of the same service where they received speech therapy and otorhinolaryngology care.

The European Organization for Research and Treatment of Cancer Quality of Life Questionnaire Core 30 (EORTC QLQ-C30) was used to characterize quality of life. This instrument assesses aspects of health, treatment, and disease of cancer patients. It consists of 30 items, with questions that address functional aspects, symptoms, quality of life, financial impact, and general health. The first 28 items use a 4-point Likert scale (1 = none, 2 = little, 3 = moderate, 4 = very much) and the last 2 items use a 7-point Likert scale (ranging from 1 = very bad to 7 = excellent). In the EORTC QLQ-30, higher scores on the overall and functional health scales indicate better quality of life. On the symptom scale, higher scores indicate worse quality of life.

Data were analyzed using descriptive statistics, calculating means and standard deviations of the scores of studied protocols. The normality of the data was tested by Shapiro-Wilk and with the non-normal distribution attested, the Mann-Whitney test was applied (*p* < 0.05 adopted) to verify differences in the scores of the protocols used considering the group at risk for dysphagia and the group without risk for dysphagia (tracked by applying the EAT-10). The Spearman’s correlation test (considering *p*-value < 0.05) was used to find relations between the domains of such protocols. Regarding the values of the correlations, values that were closer to the extremes (−1 or 1) indicated stronger correlations. Values closer to 0 indicated weaker correlations.

## Results

In this study, the final sample consisted of 12 laryngectomized subjects, 91.6% (n = 11) were male subjects and 8.4% (n = 1) were female subjects, with an average age of 64-years.

Table 1 shows the sample characteristics regarding age, sex, year of diagnosis, start of speech therapy, and type of rehabilitation and treatment.

**Table 1:** Sample characterization.

Subject	Age	Sex	Year of diagnosis	Year of speech therapy	Rehabilitation	Treatment
1	52	M	2018	2020	NA	CRT
2	63	M	2017	2019	NA	IODINE
3	62	M	2011	2017	TV	RDT
4	68	M	2013	2013	TV	CRT
5	76	M	2015	2015	TV	RDT
6	61	M	2010	2010	TV	CRT
7	56	M	2019	2019	NA	CRT
8	66	M	2015	2016	TV	CRT
9	64	F	2013	2015	EV	CRT
10	64	M	NA	NA	TV	CRT
11	75	M	2015	2015	TV	CRT
12	66	M	2013	2013	TV	NA

TV, Tracheoesophageal Voice; EV, Esophageal Voice; CRT, Chemoradiotherapy; RDT, Radiotherapy; NA, Not Applicable.

Of all participants, 67% (n = 8) used a voice prosthesis to produce tracheoesophageal voice, 8% (n = 1) communicated using the esophageal voice technique, and 25% (n = 8) used articulated speech or another type of communication, such as writing.

Regarding the type of treatment, 67% (n = 8) of the individuals underwent chemoradiotherapy, 16.4% (n = 2) underwent radioactive iodine therapy after laryngectomy, while 8.3% (n = 1) underwent radioiodine therapy. Also, one subject underwent total laryngectomy exclusively. In this study, the average time from laryngectomy to data collection ranged from 24 to 120 months.

Among the temporary or permanent side effects observed among cancer patients (odynophagia, trismus, xerostomia, mucositis, anosmia, ageusia, and intermittent cough), 92% (n = 11) of the subjects presented two or more side effects, while only 8% (n = 1) presented one of these effects.

Table 2 shows the results obtained in VoiSS and EAT-10 protocols.

**Table 2:** Results obtained from the application of the VoiSS and EAT-10 protocols.

Subject	Voice symptom scale (VoiSS)				EAT-10
	Total	Limitation	Emotional	Physical	
1	54	35	21	2	3
2	33	30	3	0	0
3	39	34	0	5	2
4	79	45	27	7	24
5	74	38	28	8	8
6	20	20	0	0	8
7	93	43	32	18	13
8	42	32	0	10	3
9	102	50	26	26	30
10	44	14	26	4	2
11	90	48	19	23	4
12	44	30	10	4	0
Mean	60	35	16	9	8
SD	26.88	10.84	12.53	8.78	9.68

All subjects exceeded the VoiSS cutoff score and in the EAT-10, 66.7% (n = 8) were at risk for dysphagia, and of these, all were subjected to at least radiation during the treatment.

Table 3 presents the scores obtained in the SRQ-20 and in the EORTC-QLQ30. The score obtained in the SRQ-20 indicated that 33.3% (n = 4) of all participants presented emotional distress.

**Table 3:** Results obtained from the application of the EORTC-C30 and SRQ-20 protocols.

Subject	EORTC-C30			SRQ-20
	Functional	General QoL	Symptms	
1	62.2	66.7	2.6	6
2	86.7	83.3	0.0	3
3	97.8	100.0	2.6	0
4	57.8	33.3	43.6	10
5	97.8	75.0	7.7	3
6	86.7	91.7	0.0	0
7	51.1	83.3	30.8	5
8	93.3	50.0	2.6	1
9	44.4	33.3	87.2	18
10	66.7	100.0	7.7	9
11	40.0	41.7	76.9	13
12	73.3	75.0	7.7	5
Mean	71	69	22	6
SD	20.80	24.45	30.93	5.52

The results obtained in each of the instruments and their domains were correlated using the Spearman’s correlation test. Table 4 shows the outcomes obtained.

**Table 4:** Correlation values between the scores obtained from the protocols using the Spearman correlation test.

	VoiSS-General	VoiSS-F	VoiSS-L	VoiSS-E	SRQ-20	EAT-10
SRQ-20	0.794^a^	0.478	0.585^a^	0.592^a^	−	−
EAT-10	0.663^a^	0.590^a^	0.705^a^	0.549	0.381	−
EORTC-F	−0.742^a^	−0.451	−0.575	−0.471	0.868^a^	0.459
EORTC-S	−0.624^a^	−0.564	−0.737	−0.266	0.625^a^	0.523
EORTC-G	0.922a	−0.809^a^	0.760^a^	0.670^a^	0.825^a^	0.583^a^

a *p*-value < 0.05; VoiSS-F, Functional domain; VoiSS-L, Limitation domain; VoiSS-E, Emotional domain; EORTC-F, Functional domain; EORTC-S, Symptom domain; EORTC-G, General domain.

Table 5 shows the comparison between the mean scores obtained from the Voice Symptom Scale (VoiSS) and the EORTC QLQ-30 among subjects with and without risk for dysphagia, as indicated in the Eating Assessment Tool (EAT-10). In all aspects assessed on the Voice Symptom Scale (VoiSS), subjects at risk for dysphagia indicate a greater presence of symptoms when compared to those without a risk for dysphagia, but a statistical difference was attested at the Limitation domain. In the EORTC QLQ-30 there is statistical difference between groups in the General Health and Quality of Life domain. In this protocol higher scores on the overall and functional health scales indicated better quality of life, while on the symptom scale, higher scores indicated worse quality of life.

**Table 5:** Comparison of the risk for dysphagia and VoiSS and the EORT-C QLQ-30 based on the mean scores calculated for subjects with and without risk for dysphagia and the p-valor considering Mann-Whitney test to reach differences between the groups.

	Voice Symptom Scale (VoiSS)			
	General level	Limitation	Emotional	Physical
EAT-10 without risk for dysphagia	41.5	30	6.5	4
EAT-10 with risk for dysphagia	69.25	38.87	19.12	9
*P*-valor (Mann-Whitney)	0.106	0.005^a^	0.304	0.125

	European Organization for Research and Treatment of Cancer Quality of Life Questionnaire Core 30 (EORTC QLQ-C30)		
	Functional Scale	General health and quality of life	Symptoms
EAT-10 without risk for dysphagia	81.11	89.58	4.49
EAT-10 with risk for dysphagia	66.67	59.38	31.41
*P*-valor (Mann-Whitney)	0.268	0.049^a^	0.3

a *p*-valor < 0.05: statistical difference.

## Discussion

The population affected by head and neck cancer presents acute and/or late effects as a result of the cancer treatment, with the potential to affect the voice, swallowing, quality of life and mental health of these subjects.^19^ In order to understand the relationships between these aspects, specific protocols for each domain were applied in this study in order to assess possible correlations between the scores of these instruments.

The sample consisted of 12 subjects who had undergone total laryngectomy, mostly male patients with an average age of 64 years. This profile is consistent with that found in other studies conducted with the same population, showing a predominance of laryngeal cancer in male subjects aged over 60 years.^20^ Regarding the type of treatment and speech rehabilitation, adjuvant chemoradiotherapy after total laryngectomy and the use of a tracheoesophageal prosthesis were the most common.

Oliveira and Marialva (2017)^21^ also found a predominance of the use of tracheoesophageal prosthesis as a form of speech rehabilitation in their study. Studies conducted with individuals who use tracheoesophageal voice show this population has a similar quality of life to those who undergo chemoradiotherapy alone, that is, with a preserved phonatory system.^22^

Speech therapy follow-up has been described in the literature as essential, with benefits in the rehabilitation of laryngectomized patients before and after treatment, mainly related to swallowing.^23^ However, 42% of the individuals in this study had no pre-treatment intervention and required at least 12-months after surgery to begin follow-up with the speech therapist, highlighting the possible impact of this late onset of intervention.

Regarding voice aspects, all subjects in the sample exceeded the VoiSS cutoff score, thus indicating self-perceived voice impairment. In a previous study,^15^ the VoiSS was applied to 96 subjects; of these, 48 were in the experimental group, treated for head and neck cancer and presenting swallowing complaints, followed by a diagnosis of dysphagia through videofluoroscopy. The values were, on average, lower than those obtained in our study, despite the similarity in the physical scale. This difference can be attributed to the smaller number of participants in our sample, as well as the fact that the voice complaint in the group studied by Moreti et al. (2018)^15^ was not presented as the main complaint, but rather swallowing changes.

Intervention to reestablish communication is essential, since studies show that subjects after laryngectomy undergoing speech therapy show improvement in the communication domain and, consequently, in health and quality of life.^24^

In Table 4, when the outcomes of the quality of life instruments and voice symptoms are correlated, it can be seen that the values obtained in the “EORTC QLQ-C30 General” and “VoiSS ‒ General domain and Limitations” show a positive correlation, indicating that the better the quality of life, the greater the self-reported voice symptoms. On careful analysis, we observed that the Global domain of the EORTC QLQ-C30 instrument has only two broad questions: “How would you rate your general health during the last week?” and “How would you rate your overall quality of life during the last week”. On the other hand, the VoISS is an specific instrument which, in both the General and Limitations domains, presents aspects intrinsically related to voice aspects, justifying the disparities observed.

The negative correlation between the “Symptoms” domain of the EORTC-S and all the outcomes obtained in the VoiSS is also justified by the same perspective. When analyzing the issues addressed in the first instrument, these issues referred to fatigue, pain, insomnia, nausea, and queasiness, not considering voice aspects.

Regarding self-perceived voice impairment in subjects with and without risk for dysphagia, the population with swallowing complaints presented a higher incidence of voice symptoms, in agreement with prior studies with this population.^9^, ^15^ Voice and swallowing have close anatomical structures, allowing for the occurrence of simultaneous impairments.^9^, ^15^ Therefore, it is extremely important to analyze voice aspects and the risk for dysphagia simultaneously, given their relevance to the quality of life of total laryngectomy subjects and the high likelihood of concomitant changes.

Oropharyngeal dysphagia has emerged as a frequent symptom resulting from the impact of surgery and adjuvant treatment modalities, used individually or simultaneously.^25^ In our study, the majority of participants were at risk of dysphagia (66.7%), and these individuals had a poorer quality of life, greater functional and health impairments, as well as marked vocal symptoms when compared to non-dysphagic individuals.

The risk of dysphagia is a factor that contributes to the worsening quality of life in total laryngectomized patients, highlighting the importance of early evaluation of swallowing in these individuals to provide adequate rehabilitation based on their complaints and thus minimize the impact of swallowing changes on the quality of life of these individuals.^26^

The inferential analysis showed positive correlation between the SRQ-20 and all the outcomes obtained in the other instruments (ESV, EAT-10 and EORTC QLQ-C30) (Table 4), showing that in those individuals who presented psychological distress, we observed a greater risk of dysphagia and vocal symptoms, as well as a worse quality of life.

Individuals who have undergone total laryngectomy face internal conflicts and psychological impacts from the moment of diagnosis, varying according to the particularities of each case (location of the tumor, treatment modality and stage of cancer).^27^ It is essential that discussions on the Tumor Board considers the psychological aspects of decision-making, given the evidence that suggests the imminent impact of cancer treatment on the mental health and quality of life of these individuals. It is essential that health services for the cancer population have qualified professionals who are attentive to the psychological suffering of these individuals, in order to provide appropriate follow-up whenever necessary.

The concept of quality of life is multifaceted as it considers the unique aspects of each individual, varying according to the emotional, physical, religious, financial and/or other domains that are extremely important to the individual, making it difficult to establish objective metrics. Despite these challenges, measuring the impact of cancer treatment on the quality of life of laryngectomized patients receiving speech therapy and multidisciplinary follow-up supports appropriate rehabilitation, as seen with comprehensive health care for this population, which improves the biopsychosocial well-being of these individuals.

Future studies should further explore the topic discussed here, including a more comprehensive sample, since our sample size was a study limitation, which prevents the generalization of the results to other groups of subjects with total laryngectomy than those included in this study. However, we highlight that studies involving this population are scarce and with limited number of participants, in addition to the fact that this study was conducted during the COVID-19 pandemic period, even with such limitations, the present study becomes relevant as it contributes to the literature and to increase the knowledge in this vulnerable population. Healthcare teams need to be aware of the importance of having a psychologist in the healthcare team for this population, aiming to deeply address the aspects related to mental health in laryngectomized patients.

## Conclusion

The study sample consisted mainly of male patients with an average age of 64 years. Tracheoesophageal prosthesis was the most common method of communication. All subjects had voice symptoms.

Risk for dysphagia is as a determinant of quality of life and mental health in the study group, also related to the concomitance of voice symptoms.

In our sample, patients with a high prevalence of voice signs and symptoms had a higher risk of emotional distress.

## Financing

Institutional Scientific Initiation Scholarship Program – PIBIC of UNICAMP.

## Declaration of competing interest

The authors declare no have conflicts of interest.
